# The Possible Effect of B-Cell Epitopes of Epstein–Barr Virus Early Antigen, Membrane Antigen, Latent Membrane Protein-1, and -2A on Systemic Lupus Erythematosus

**DOI:** 10.3389/fimmu.2018.00187

**Published:** 2018-02-12

**Authors:** Jianxin Tu, Xiaobing Wang, Guannan Geng, Xiangyang Xue, Xiangyang Lin, Xiaochun Zhu, Li Sun

**Affiliations:** ^1^Department of Rheumatology, The First Affiliated Hospital of Wenzhou Medical University, Wenzhou, China; ^2^Central Laboratory, Renji Hospital, Shanghai Jiao Tong University School of Medicine, Shanghai, China; ^3^Department of Microbiology and Immunology, Basic Medical College of Wenzhou Medical University, Wenzhou, China; ^4^Medical Laboratory, The First Affiliated Hospital of Wenzhou Medical University, Wenzhou, China

**Keywords:** Epstein–Barr virus, systemic lupus erythematosus, B-cell epitopes, cross-reactivity, early antigen protein D, envelope glycoprotein GP340/membrane antigen, latent membrane protein-1, latent membrane protein-2A

## Abstract

This study was aimed to evaluate the role of B-cell epitopes of Epstein–Barr virus (EBV) Early antigen protein D (EA), envelope glycoprotein GP340/membrane antigen (MA), latent membrane protein (LMP)-1, and LMP-2A in systemic lupus erythematosus (SLE). B-cell epitopes were predicted by analyzing secondary structure, transmembrane domains, surface properties, and homological comparison. 60 female mice were randomized equally into 12 groups: 1–10 groups were immunized by epitope peptides (EPs) 1–10, respectively, while 11 and 12 groups were PBS and Keyhole limpet hemocyanin (KLH) control groups. Immunoglobulin G (IgG) and autoantibody to nuclear antigen (ANA) concentrations in mice serum were determined at week 8. Indirect levels of EP1–10 were further detected by enzyme-linked immuno sorbent assay (ELISA) in 119 SLE patients and 64 age- and gender-matched health controls (HCs). 10 probable EBV EA, MA, LMP-1, and LMP-2A B-cell epitopes related to SLE self-antigens were predicted and corresponding EP1–10 were synthesized. IgG concentrations at week 8 were increased in EP1–10 and KLH groups compared with PBS group in mice; while ANA levels were elevated in only EP1–4, EP6–7, and EP10 groups compared to KLH group by ELISA, and ANA-positive rates were increased in only EP1, EP2, EP4, EP6, and EP10 groups by indirect immunofluorescence assay. EP1–4, EP6, and EP10 indirect levels were increased in SLE patients than HCs, while EP1, EP3, EP6, and EP9 were correlated with SLE disease activity index score. In conclusion, EBV EA, MA, LMP-1, and LMP-2A B-cell EPs increased SLE-related autoantibodies in mice, and their indirect levels might be served as potential biomarkers for SLE diagnosis and disease severity.

## Introduction

Systemic lupus erythematosus (SLE) is a chronic inflammatory autoimmune disease which is characterized by dysregulated autoantibodies and affected organs, including kidney, skin, joint, central nervous system, and so on (1). SLE mostly affects women of childbearing age, and its incidence as well as prevalence keeps increasing worldwide in recent decades (2). 5.2 per 10,000 populations in the US suffer from SLE, while 2.6 and 2.8 per 10,000 populations in the UK and Japan have SLE, respectively; as to China, the prevalence is 3.7 per 10,000 populations (2, 3). SLE patients present with a lot of clinical phenotypes of systemic autoimmunity, among which half have arthritis, a third have malar rash, and 28% have active nephropathy (4, 5). Although the pathogenesis of SLE is still ambiguous, genetic susceptibility, environmental factors, and disturbances in both innate and adaptive immunity are considered to be involved in SLE etiology (1).

Among environmental factors of SLE etiology, Epstein–Barr virus (EBV) is regarded as one of the possible triggers (6–9). Accumulating evidences report that SLE patients present with an elevated EBV-infected rate compared with age-matched controls, and antibodies against EBV antigens are also increased in SLE patients (10, 11). By application of quantitative polymerase chain reaction (PCR), an increase of 15- to 40-fold of EBV load in peripheral blood mononuclear cells (PBMCs) is discovered in SLE patients than health controls (HCs) (12, 13). And four EBV viral mRNAs [BamHI Z leftward open reading frame (BZLF)-1, latent membrane protein (LMP)-1, LMP-2, and nuclear antigen of EBV (EBNA)-1] are illuminated to be abnormally expressed in PBMCs from SLE patients than controls (14). Besides, SLE patients disclose raised EBV-infected peripheral B cells which are independent of intake of immunosuppressive medication and elevated concentration of EBV DNA copy count compared to healthy controls, and the frequency of infected B cells is observed to be positively correlated with disease severity in SLE patients (14, 15). In addition, EBV-induced infectious mononucleosis (IM) shares similar symptoms and clinical manifestations as SLE, and the presence of rheumafactor and autoantibodies against cellular components such as DNA, histones, and ribonucleoproteins are detectable in both EBV-induced IM patients and SLE patients (16, 17). These indicate the possibly important role of EBV in SLE etiology. However, the mechanism of how EBV function in SLE pathogenesis is still obscure. Some evidences suggest that infection and immortalization of autoreactive B-cells, T-cells, and NK cells; exacerbated inflammation; activation of human endogenous retroviruses (HERVs); cross-reactivity between microbial peptides and similar self-peptides; and augmenting autoimmunity through bystander activation may contribute to the cause of EBV for SLE development and progression (18–25).

Molecular mimicry, as proposed by Fujinami et al. for the first time, might be one of the main hypotheses on how EBV infection causes autoimmunity, which is the concept that sequential and/or structural similarities between microbial peptides and self-peptides can allow expansion of microbial specific T-cells and B-cells which stimulate cross-reactivity to similar self-peptides (18, 26). Cross-reactivity of autoantibodies against epitopes on SLE small nuclear ribonucleoprotein-associated protein B (SmB) and SmD with various domains of EBNA-1 has been reported in SLE, and lupus-like autoimmune disease appears in rabbits after immunization by EBNA-1 motif PPPGRRP (27–29). While being immunized by the entire EBNV-1 protein, the development of serum anti-dsDNA and anti-Sm antibodies in mice is observed (30). These indicate molecular mimicry plays critical role in the connection of EBV infection with SLE etiology.

Early antigen protein D (EA), envelope glycoprotein GP340/membrane antigen (MA), LMP-1, and LMP-2A are essential parts of EBV and have been observed to be correlated with SLE susceptibility (6, 31, 32), And our preliminary investigation found that EBV EA, MA, LMP-1, and LMP-2A mRNAs expressions were elevated in PBMCs of SLE patients, and previous reports by our lab disclosed that the B epitopes of LMP-2 presented with good antigenicity and immunogenicity in mice (33, 34). However, how EBV EA, MA, LMP-1, and LMP-2A function in the mechanism of SLE is still needed to be further understood. This study was aimed to predict B-cell epitopes of EBV EA, MA, LMP-1, LMP-2A related to SLE self-antigens, combine and purify B-cell epitope peptides (EPs) and to investigate their immunogenicity and antigenicity through animal experiments, and further evaluate the association of serum EPs indirect levels with risk and disease severity in SLE patients.

## Materials and Methods

### Prediction of EBV EA, MA, LMP-1, and LMP-2A B-Cell Epitopes

The complete amino acid sequences of EBV EA, MA, LMP-1, and LMP-2A were retrieved from UniProKB Protein Database in Swiss Institute of Bioinformatics^1^. And the accession number and accession name on UniProKB Protein Database for each protein investigated in this study was as follows: (1) EBV EA, accession number A0A191T7H7, accession name A0A191T7H7_EBVG; (2) EBV MA, accession number P03200, accession name GP350_EBVB9; (3) EBV LMP-1, accession number P03230, accession name LMP1_EBVB9; and (4) EBV LMP-2A, accession number P13285, accession name LMP-2_EBVB9. The secondary structure prediction of the EBV EA, MA, LMP-1, and LMP-2A was analyzed by Protean module in DNAStar software^2^ as follows: (1) created a file for candidate sequence and (2) chose the following items: Amphiphilicity-Eisenberg; Secondary structure-Garnier-Robson; Secondary structure-Chou-Fasman. Transmembrane domains were specified using TMpred module on EXPASY Internet Server^3^ as follows: (1) set output format, (2) chose input sequence format as plain text and entered the protein sequence, and (3) suggested model for transmembrane topology was required.

And surface properties of EBV EA, MA, LMP-1, and LMP-2A proteins were analyzed as well by various modules in DNAStar software (see text footnote 2) as follows: (1) Created a file for candidate sequence and (2) Chose the following items: Surface probability-Emini (for detection of features of probability); Flexibility–Karplus-Schulz (for detection of features of flexibility); Hydropathy–Kyte-Doolittle (for detection of features of hydrophilicity); Antigenicity–Jameson-Wolf (for detection of features of antigenicity). In addition, Polarity was analyzed by ProtScale module on EXPASY Internet Server^4^ as follows: (1) entered the protein sequence and (2) chose scale Palarity/Zimmerman.

According to the results of above analysis, B-cell epitopes of EBV EA, MA, LMP-1, and LMP-2A with good probability, flexibility, strong antigenicity, and hydrophilicity were identified. And BLAST module in NTI8.0 Vector Software was used to compare the amino acid sequences of EBV EA, MA, LMP-1, and LMP-2A B-cell epitopes and SLE self-antigens [Sm B, Sm D, Sm E, ribonucleoprotein (rRNP), and Sjogren’s syndrome A (SSA/Ro)] to acquire the similarity rate, in order to subsequently identify the B-cell epitopes with high homology to SLE self-antigens. Despite of similarity rate between EBV epitopes and SLE self-antigen epitopes, we also determined the features of other different amino acids by analyzing their hydrophilicities and acid–base properties. And “percentage of amino acids with similar characteristics” was calculated, which was defined as the rate of amino acids with similar hydrophilicity or acid–base property between EBV epitopes and SLE self-antigens, which revealed the homology between EBV and SLE epitopes to some extent.

### Combination and Purification of EPs

Selected SLE related EBV EA, MA, LMP-1, and LMP-2A B-cell EPs were combined by a bio-technology company (Zexiyuan Bio-Tech Company, China). EPs were purified and determined by Delta 600 HPLC, Mass Spectrometer was used to measure molecular weight of peptides. EPs with purification above 95% were subsequently stored at −20°C for further experiments. Besides, Keyhole limpet hemocyanin (KLH) was used as immunological carrier bounded with purified EPs for animal experiment in next steps (35, 36).

### Mice Immunization and Serum Sample Collection

60 female specific pathogen free C57/6j mice aged 6 weeks were randomized equally into 12 groups: EP1–10 groups as experimental groups, while PBS group and KLH group as controls, with five mice in each group. The mice in EP1 group to EP 10 group were subcutaneously inoculated by 100 µg purified EPs and KLH at weeks 1, 3, and 5, while 100 µg PBS and KLH were performed in PBS group and KLH group, respectively. All antigens were emulsified with an equal volume of Freund’s incomplete adjuvant just before injection, except for the first dose with Freund’s complete adjuvant. Tail blood was collected and serum was separated at week 8 and stored at −80°C.

The mice experiment has been approved by the Animal Ethics Committee of the First Affiliated Hospital of Wenzhou Medical University, and all related experiments were conducted according to the “Code for the Care and Use of Animals for Scientific Purposes” statement and under the principles of 3R (replacing, refining, and reducing).

### Determination of Immunogenicity by Immunoglobulin G (IgG) Measurement in Mice Serum by Enzyme-Linked Immunosorbent Assay (ELISA)

Serum IgG level in mice serum of EP1–10 group, KLH control group, and PBS control group was detected by ELISA to determine the immunogenicity as follows: Anti-mouse IgG antibody (Abcam, USA) were coated in the 96-well microplates, then mice serum samples were diluted as 1:1,000 ratio as primary antibody and sealed with 5% skim milk, subsequently 1:2,000 diluent goat anti-mouse IgG-HRP (Abcam, USA) was added as secondary antibody; After incubation with TMB (TIANGEN, China), the absorbance (OD) was then measured at 450 nm by using a Bio-Tek ELISA microplate reader. Concentration was calculated by OD and standard curve (derived from measurement of standard substance). Standard substance was provided by Solarbio company (Solarbio, China). All samples were independently analyzed in triplicate, and the mean value of concentrations was used for analysis in this study.

### Determination of Antigenicity by Autoantibody to Nuclear Antigen (ANA) Measurement in Mice Serum by ELISA and Indirect Immunofluorescence Assay

Autoantibody to nuclear antigen in mice serum sample of each group was measured by commercial ELISA kit (Solarbio, China) according to the instructions of manufacturer to determine the antigenicity of EP1–EP 10. In addition, indirect immunofluorescence assay was performed using EUROPLUS ANA Mosaic 20A kit (Euroimmun, German) according to the instructions of manufacturer as follows: Serum samples from mice were pooled and diluted at 1:40, 1:80, 1:160, 1:320, and 1:640 with PBST, and incubated with Hep-2 cells smear on slides for 30 min. The slides were then incubated with 1:100 FITC-conjugated rabbit anti-mouse IgG after washing. The slides were subsequently screened using fluorescence microscopy (Nikon, Japan) after washing. Known mouse ANA-positive serum (BXSB mouse) and mouse ANA-negative serum (healthy C57/6j mouse) with FITC-conjugated rabbit anti-mouse IgG were served as the positive control and negative control, respectively. Two independent experts were invited to analyze the expression of ANA by indirect immunofluorescence assay, and samples were classified as ANA positive if a well-defined indirect immunofluorescence pattern was identified at 1:80 dilution by both two observers according to the method described in previous study (37).

### Determination of Antigenicity by Anti-SmB, Anti-SmD, Anti-SmE, Anti-rRNP, and Anti SAA/Ro Antibodies Measurement in Mice Serum Using ELISA

Expression of anti-extractable nuclear antigens (ENAs) antibodies, including anti-SmB antibody, anti-SmD antibody, anti-SmE antibody, anti-rRNP antibody and anti-SSA/Ro antibody, in mice serum samples from each group were measured using commercial ELISA kits (Solarbio, China) according to the instructions of manufacturer to determine the antigenicity of EP1 to EP10.

### Participants

In order to further investigate the role of EBV EA, MA, LMP-1, and LMP-2A B-cell EPs in SLE patients, a total of 119 SLE patients were consecutively recruited in this study from February 2016 to December 2016 at Department of Rheumatology in the First Affiliated Hospital of Wenzhou Medical University. All patients were diagnosed with SLE according to 1982 American College of Rheumatology (ACR) criteria for the classification of SLE. 64 age- and gender-matched HCs were enrolled in the same duration at Department of Physical Examination as well. HCs with history of rheumatoid diseases, severe infection, malignant tumors, and severe hepatic or renal dysfunction were excluded. All participants provided written informed consents, and the Ethics Committee of the First Affiliated Hospital of Wenzhou Medical University approved this study.

### Serum Sample Collection in SLE Patients and Indirect ELISA Analysis

Serum samples were collected from all SLE patients and HCs. 100 µl EP (10 µg/mL) was coated in each microplate. 1:50 diluted serum sample was used as primary antibody and sealed with 5% skim milk, while 1:10,000 diluent goat anti-human IgG-HRP (Abcam, USA) was applied as secondary antibody to carry out indirect ELISA. Post being incubated with DAB (TIANGEN, China), OD was subsequently measured at 450 nm by using a Bio-Tek ELISA microplate reader. All samples were independently analyzed in triplicate, and the mean value of OD was used for analysis in this study.

### Disease Severity Assessment

SLE disease activity index (SLEDAI) score was calculated according to the scale of SLEDAI-2K (38) in SLE patients to evaluate the disease severity so as to explore the correlation of serum EPs levels with disease severity.

### Statistics

SPSS 21.0 Software (Chicago, IL, USA) and GraphPad 5.0.1 software (GraphPad, USA) were used for statistical analysis. Data were mainly presented as mean ± SD, median (1/4–3/4), or count (%). Comparison between two groups was determined by *t* test, Wilcoxon rank sum test, or Chi-square test. Bonferroni correction was performed in multiple tests for comparison of EP levels between SLE patients and HCs. Receiver Operating Characteristic (ROC) curve was drawn to evaluate the predictive value of B-cell EPs for predicting SLE risk. Spearman rank correlation test was used to analyze the correlation of serum EPs levels with disease severity in SLE patients. *P* value < 0.05 was considered significant.

## Results

### Secondary Structure Prediction of EBV EA, MA, LMP-1, and LMP-2A

Protean module of DNAstar software was used to predict the secondary structure of EBV EA, MA, LMP-1, and LMP-2A, which revealed: (1) EBV EA mainly consisted of β sheets, followed by α helixes, and some inconstant coils as well as β turns (Figure S1A in Supplementary Material). Inconstant coils were mostly located at: 43–45, 64–67, 73–79, 120–122, 337–341, 349–352, 355–357, 362–367, and 381–383 near the N-terminal. (2) EBV MA mostly contained β sheets, followed by inconstant coils, β turns, and fewer α helixes (Figure S1B in Supplementary Material). Inconstant coils were mostly located at: 22–25, 82–89, 116–119, 203–206, 309–318, 353–357, 395–399, 656–660, 674–679, 697–702, 710–722, 729–732, 770–775, and 814–818 near the N-terminal. (3) EBV LMP-1 mainly included β sheets, followed by α helixes, β turns, and inconstant coils (Figure S1C in Supplementary Material). Inconstant coils were mostly located at 8–12, 189–193, 202–206, 211–216, 247–257, 310–312, 320–324, and 336–338 near the N-terminal. (4) EBV LMP-2A was mainly made up by α helixes, followed by inconstant coils, and some β sheets as well as β turns (Figure S1D in Supplementary Material). Inconstant coils were mostly located at: 9–29, 33–80, 89–108, 201–206, 289–294, 339–346, 380–388, and 484–492 near the N-terminal.

### Transmembrane Domains Analysis

TMpred in EXPASY was applied to analyze the transmembrane domains of EBV EA, MA, LMP-1, and LMP-2A, which disclosed that EBV EA had three predicted transmembrane domains located at 47–65, 240–265 and 267–285 (Figure S2A in Supplementary Material), EBV MA had six predicted transmembrane domains located at 1–19, 90–106, 244–260, 274–295, 381–406, and 840–860 (Figure S2B in Supplementary Material), EBV LMP-1 had 7 predicted transmembrane domains located at 25–44, 51–72, 76–95, 113–133, 142–163, 169–188, and 345–365 (Figure S2C in Supplementary Material), and EBV LMP-2A had 10 predicted transmembrane domains located at 148–168, 178–198, 217–235, 242–263, 267–288, 300–316, 323–339, 355–376, 392–410, and 450–466 (Figure S2D in Supplementary Material).

### Surface Properties of EBV EA, MA, LMP-1, and LMP-2A

Surface features of probability, flexibility, hydrophilicity, antigenicity, and polarity of EBV EA, MA, LMP-1, and LMP-2A were presented in Figures S3A–D in Supplementary Material, and possible B-cell epitopes with good probability, flexible, strong antigenicity, and hydrophilicity were analyzed.

Followed by analysis of secondary structure, transmembrane domains, surface properties of EBV EA, MA, LMP-1 and LMP-2A, 7 regions for EA, 24 regions for MA, 7 regions for LMP-1 and 10 regions for LMP-2A were predicted as B-cell epitopes which were presented in Table 1.

**Table 1:** B-cell epitopes prediction by various methods.

EBV proteins	Locations of predicted epitopes
EA	EVSPDAVAEWQNHQS (62–76), YKRPQGGSRPEF (113–125), MPPASDRLRNEQMI (149–162), WARQQGSGGVKVTLNPDLY (178–196), QDVGAVEAHVCS (230–242), ASEPEDKSPRVQPLGTGLQQRPRHTVSPSPSPPPPPRTPTWESPARPETPS (307–357), RKRTSSEARQKHPKKVKQ (379–398)
MA	IQSLI (12–16), PTCNVCTA (37–44), GKKHQLDLD (54–64), PHTKAVYQPRGAFGGSENATN (70–-90), KKLPINVTTGEEQQ (109–122), HHAEMQNPVYLIPETVPYIKWDNCNSTNI (142–170), PTSAQDSNFSVKTEMLGNEID (187–208), IMEDGEISQVLPGDNKFN (212–229), NGPKASGGDYCIQS (288–301), ASQDMPTNTTDI (311–322), TSEDANSPNVT (337–347), ATNATTTTHK (409–418), APESTTTSPTLNTT (424–437), NTTTGLPSSTHVPTNLT (443–459), TPSPSPWDNGPESKAPDMTSST (489–510), PNATSPTPVATTPTPNATS (518–536), PAVTTPTPNATSPTLGKTSPTS (539–560), TPNATSPTLGKTSPTSAVTTP (587–607), QANATNHTLGGTSPT (622–636), SAVTTGQHNITSSSTSSMS (648–666), ETLSPSTSDNSTSHMPLL (674–691), GGENITQVTPASISTHHVSTSSPAPR (698–723), ATSPQAPSGQKTAVPTVTSTG (756–776), PQAPSGQKTAVPTVTSTGGKANSTTGGKHTTGHGARTSTEPTTDYGGDSTTPRP (759–812)
LMP-1	PPGPRRPPRG (9–18), IALWNLHGQALYLGIVLFIFGC (95–116), QRHSDEHHHDDSLPHPQQATDDSGHESDSNSNEGRHHL (189–226), GAGDGPPLCSQNLGA (230–244), PQDPDNTDDNGPQDPDNTDDNGPQD (253–278), DPDNTDDNGPHDPLPH (293–308), PQLTEEVENKG (321–331)
LMP-2A	PPSPGG (13–18), PDGYDGGNNSQYPS (20–33), GNTPTPPNDEERESNEEPPPPYEDPY (39–64), NGDRHSDYQPLGTQDQ (67–82), HDG (90–92), GLPPPPYSPRDDSSQ (95–109), AAQRKLL (173–179), EDPPF (201–205), SGNRTYG (415–421), LESEERPPTPYR (483–494)

*EBV, Epstein–Barr virus; EA, early antigen protein D; MA, rnvelope glycoprotein GP340/membrane antigen; LMP, latent membrane protein*.

### Homological Analysis between EBV EA, MA, LMP-1, and LMP-2A and SLE Self-Antigens

As to determine the possible EBV EA, MA, LMP-1, and LMP-2A B-cell epitopes with high homology to SLE self-antigens, amino acid sequences of EA, MA, LMP-1, and LMP-2A were compared with SLE self-antigens, including SmB, SmD, SmE, rRNP, and Ro, and we observed 10 candidate B-cell epitopes named Epitope 1–10 as shown in Table 2. And EBV epitope 4, 6, 9, and 10 disclosed no less than 60% similarity with SLE antigen epitopes accordingly, while EBV epitope 2, 3, 7, and 8 illuminated 40 to 60% similarity (Table 2). In order to further investigate the homology between EBV epitopes and SLE epitopes, we analyzed the characteristics of other different amino acids by analyzing their hydrophilicity and acid–base property, and we found EBV epitope 4, 6, 7, 9, and 10 revealed no less of 70% amino acids with similar characteristics to SLE antigen epitopes, while EBV epitope 2, 3, 5, and 8 showed 50 to 70% (Table 2). Subsequently all EPs (EP1–10) were combined and purified according to the sequence of epitopes.

**Table 2:** 10 EBV B-cell epitopes selected by comparison with SLE pathogenic antigens.

Name	Parameters	Amino acid sequence	Similarity (%)	Percentage of amino acids with similar characteristics (%)
Epitope 1	EA (61–75)	FEVSP DAVAEWQNHQ	33	47
	rRNP	FI VGADNYGSKQMQQ		
Epitope 2	EA (114–121)	YKRPQGC S	50	63
	Ro	YKQRNGWS		
Epitope 3	EA (315–323)	PRVQPLGTG	44	67
	Sm B	PTQYPPGRG		
Epitope 4	EA (338–345)	PPPP P RTP	63	75
	Sm B	PPPGMRPP		
Epitope 5	MA (523–535)	PTPA VT TPTPN A T	36	57
	Sm B	PSQQVMTPQGRGT		
Epitope 6	LMP1 (227–236)	LVSGAGDGPP	60	70
	Sm B	LVSMTVEGPP		
Epitope 7	LMP-2A (275–283)	VSMT LL LLA	50	67
	Sm D	VSMNTHLKA		
Epitope 8	LMP-2A (344–351)	SCPLSK IL	50	75
	Sm D	SLPLDT LL		
Epitope 9	LMP-2A (291–300)	PGGLGTLGAA	60	80
	Sm B	PQGRGTVAAA		
Epitope 10	LMP-2A (156–161)	GL ALS L	67	83
	Ro	GMALAL		

*Similarity was defined as the percentage of the same amino acids between EBV proteins and SLE antigens presented in gray area. Percentage of amino acids with similar characteristics was defined as the amino acids with similar hydrophilicity or acid–base property between EBV and SLE epitopes, which revealed the homology between EBV and SLE epitopes to some extent. Hydrophilic amino acids: G, Y, N, Q, S, T, C; hydrophobic amino acids: A, V, L, I, F, W, M, P; acidic amino acids: D, E; alkaline amino acids: H, K, R*.

*EBV, Epstein–Barr virus; EA, early antigen protein D; MA, rnvelope glycoprotein GP340/membrane antigen; LMP, latent membrane protein; SLE, systemic lupus erythematosus; rRNP, ribonucleoprotein; Ro, Sjogren’s syndrome A (SSA/Ro); Sm B, Smith B; Sm D, Smith D*.

### Immunogenicity of Selected EPs by IgG Measurement in Mice Serum

**Figure 1:**
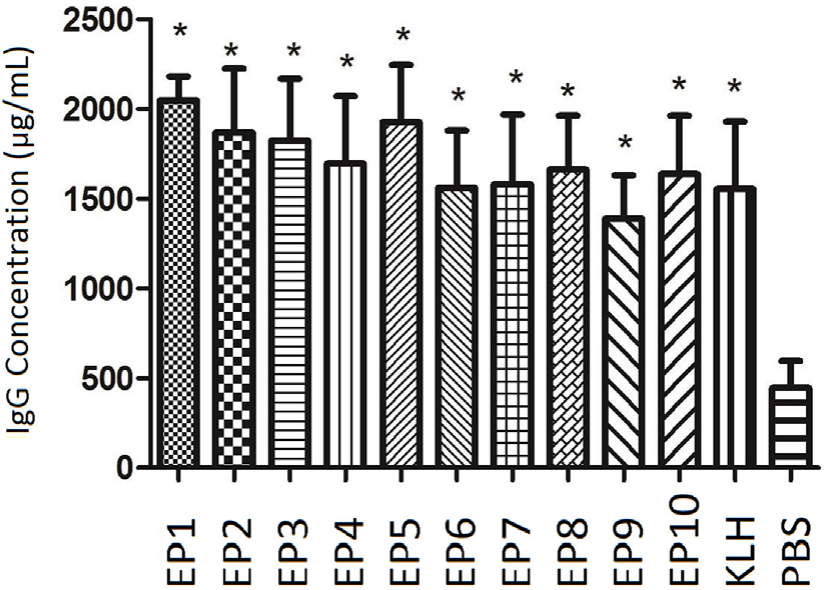
The serum Immunoglobulin G (IgG) concentration in each mice group after immunization. enzyme-linked immunosorbent assay was performed to evaluate IgG concentration in each mice group after immunization. EP1–10 and keyhole limpet hemocyanin groups presented elevated IgG levels compared with PBS group. Comparison was determined by *t* test. * vs. PBS group, *P* < 0.05.

As presented in Figure 1, IgG concentration at week 8 was dramatically increased in all EPs (EP1–10) and KLH group compared with PBS group (all *P* < 0.05) in mice experiment, which indicated all bound EPs could induce high level of IgG and presented good immunogenicity.

### Antigenicity of Selected EPs by ANA Measurement in Mice Serum

**Figure 2:**
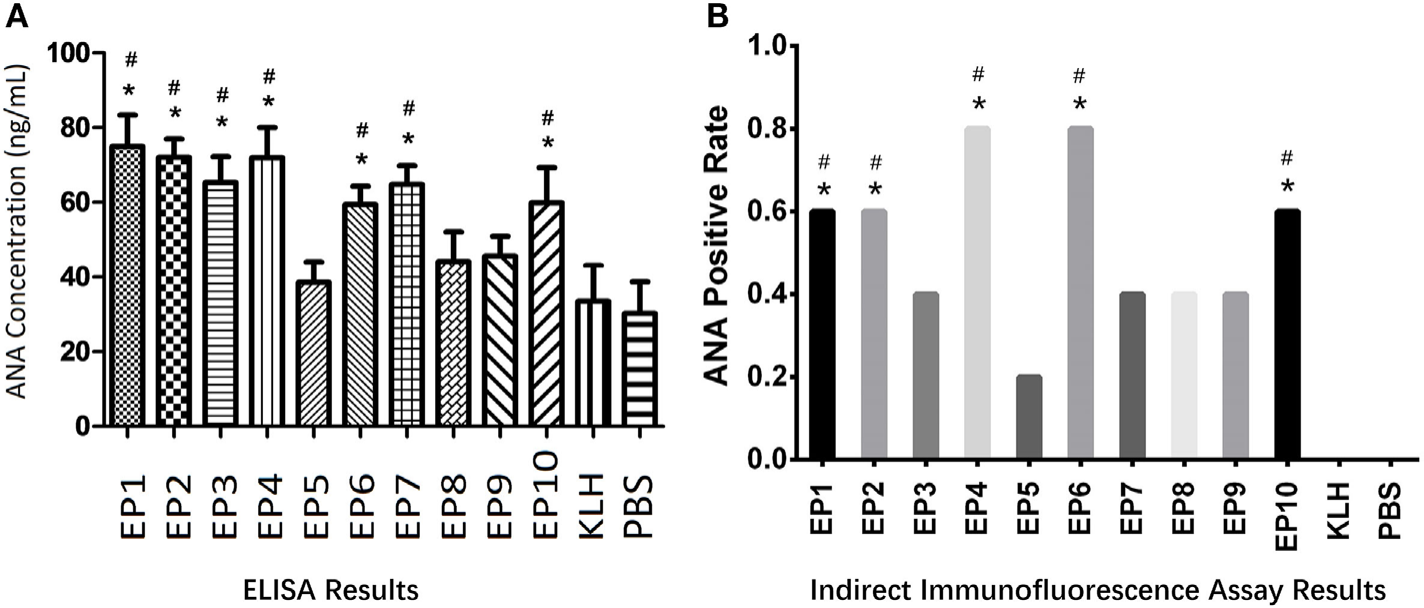
The serum autoantibody to nuclear antigen (ANA) concentration in each mice group after immunization. Enzyme-linked immunosorbent assay (ELISA) and indirect immunofluorescence assay were performed to determine the ANA expression in each group (five mice in each group) after immunization. ELISA disclosed that EP1–EP4, EP6, EP7, and EP10 groups showed increased ANA levels than keyhole limpet hemocyanin (KLH) and PBS groups **(A)**, while indirect immunofluorescence assay illuminated ANA-positive rates were elevated in EP1, EP2, EP4, EP6, and EP10 groups than KLH and PBS groups at 1:80 dilution **(B)**; Samples were classified as ANA positive if a well-defined indirect immunofluorescence pattern was identified at 1:80 dilution by both two observers according to the method described in previous study **(B)**. Comparison was determined by *t* test or Chi-square test. * vs. PBS group. *P* < 0.05, # vs. KLH group (*P* < 0.05).

Autoantibody to nuclear antigen level was detected in mice serum by ELISA as well which was presented in Figure 2A. KLH group was observed to have similar ANA concentration compared to PBS group. ANA levels in EP1, EP2, EP3, EP4, EP6, EP7, and EP10 groups were increased compared to KLH group (*P* < 0.05), while no difference was discovered in EP5, EP8, and EP9 groups compared with KLH group. Furthermore, indirect immunofluorescence assay (Figure 2B) revealed that ANA-positive rate was increased in EP1, EP2, EP4, EP6, and EP10 groups compared with KLH group (*P* < 0.05), while no difference was observed in EP3, EP5, and EP7–9 groups compared to KLH group. These suggested EP1, EP2, EP4, EP6, and EP10 might be targets of EBV in SLE pathogenesis.

### Expressions of Anti-SmB, Anti-SmD, Anti-SmE, Anti-rRNP, and Anti SAA/Ro Antibodies in Mice Serum

**Figure 3:**
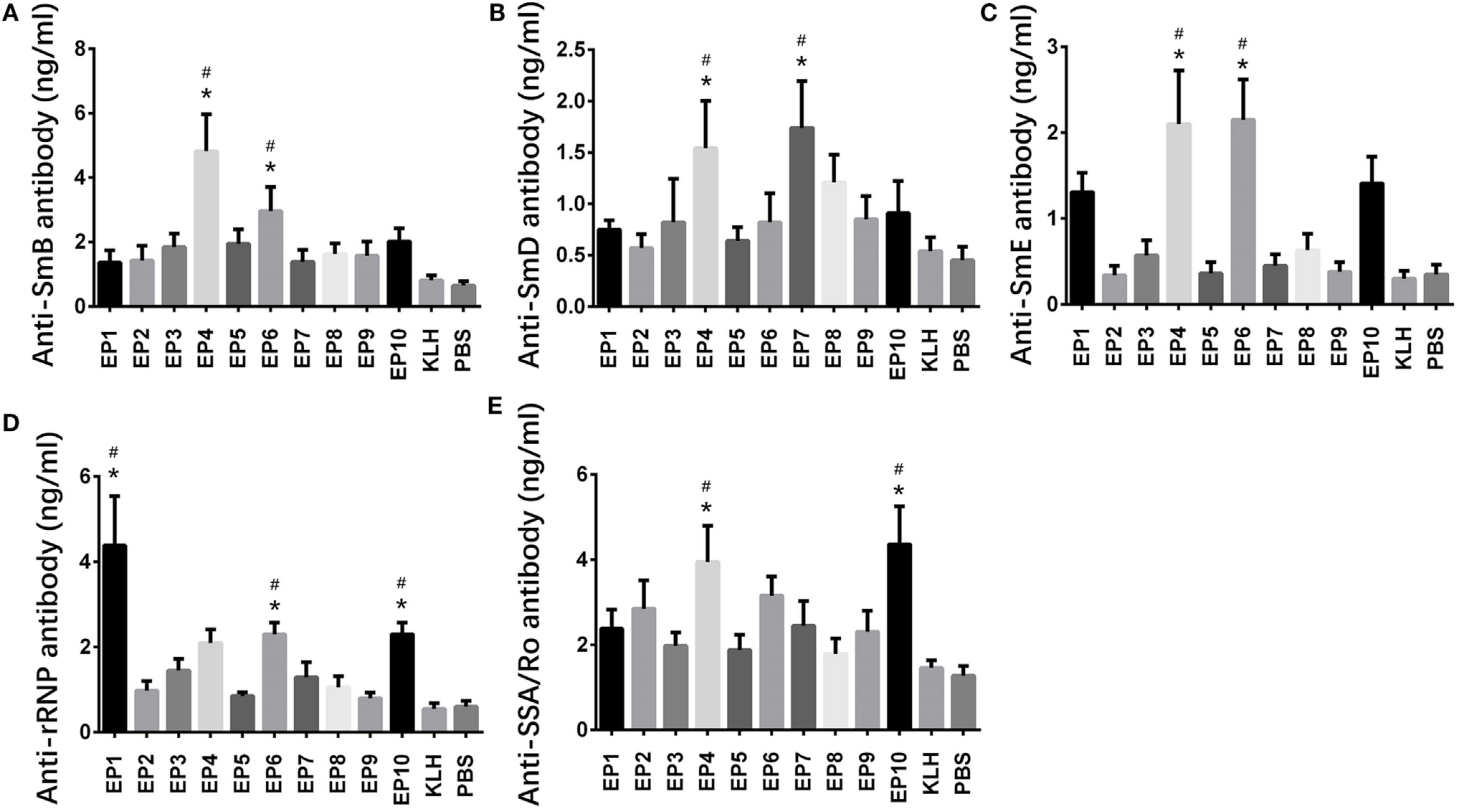
Expressions of anti-SmB, anti-SmD, anti-SmE, anti-rRNP, and anti SAA/Ro antibodies in each mice group after immunization. Enzyme-linked immunosorbent assay was performed to determine the expressions of anti-SmB, anti-SmD, anti-SmE, anti-rRNP, and anti SAA/Ro antibodies in each mice group after immunization. The results showed **(A)** elevated anti-SmB antibody level in EP4 and EP6 groups than keyhole limpet hemocyanin (KLH) and PBS groups, **(B)** elevated anti-SmD antibody level in EP4 and EP7 groups than KLH and PBS groups, **(C)** elevated anti-SmE antibody level in EP4 and EP6 groups than KLH and PBS groups, **(D)** elevated anti-rRNP antibody level in EP1, EP6, and EP10 groups than KLH and PBS groups, and **(E)** elevated anti-Sjogren’s syndrome A (SSA)/Ro antibody level in EP4 and EP10 groups than KLH and PBS groups. Comparison was determined by *t* test. * vs. PBS group. *P* < 0.05, ^#^ vs. KLH group (*P* < 0.05).

Expressions of anti-ENAs antibodies, including anti-SmB, anti-SmD, anti-SmE, anti-rRNP, and anti SAA/Ro antibodies, in mice serum were also measured by ELISA (Figure 3), which illuminated that anti-SmB antibody expression was increased in EP4 and EP6 groups compared with KLH group (Figure 3A), anti-SmD antibody expression was increased in EP4 and EP7 groups compared with KLH group (Figure 3B), anti-SmE antibody expression was elevated in EP4 and EP6 groups compared to KLH group (Figure 3C), anti-rRNP antibody expression was raised in EP1, EP6, and EP10 groups compared with KLH group (Figure 3D), while anti-SSA/Ro antibody expression was higher in EP4 and EP10 groups than KLH group (Figure 3E), these results supported the hypothesis that EP1, EP4, EP6, and EP10 might be targets of EBV in SLE pathogenesis.

### Serum EP1–10 Indirect Levels in SLE Patients and HCs

**Figure 4:**
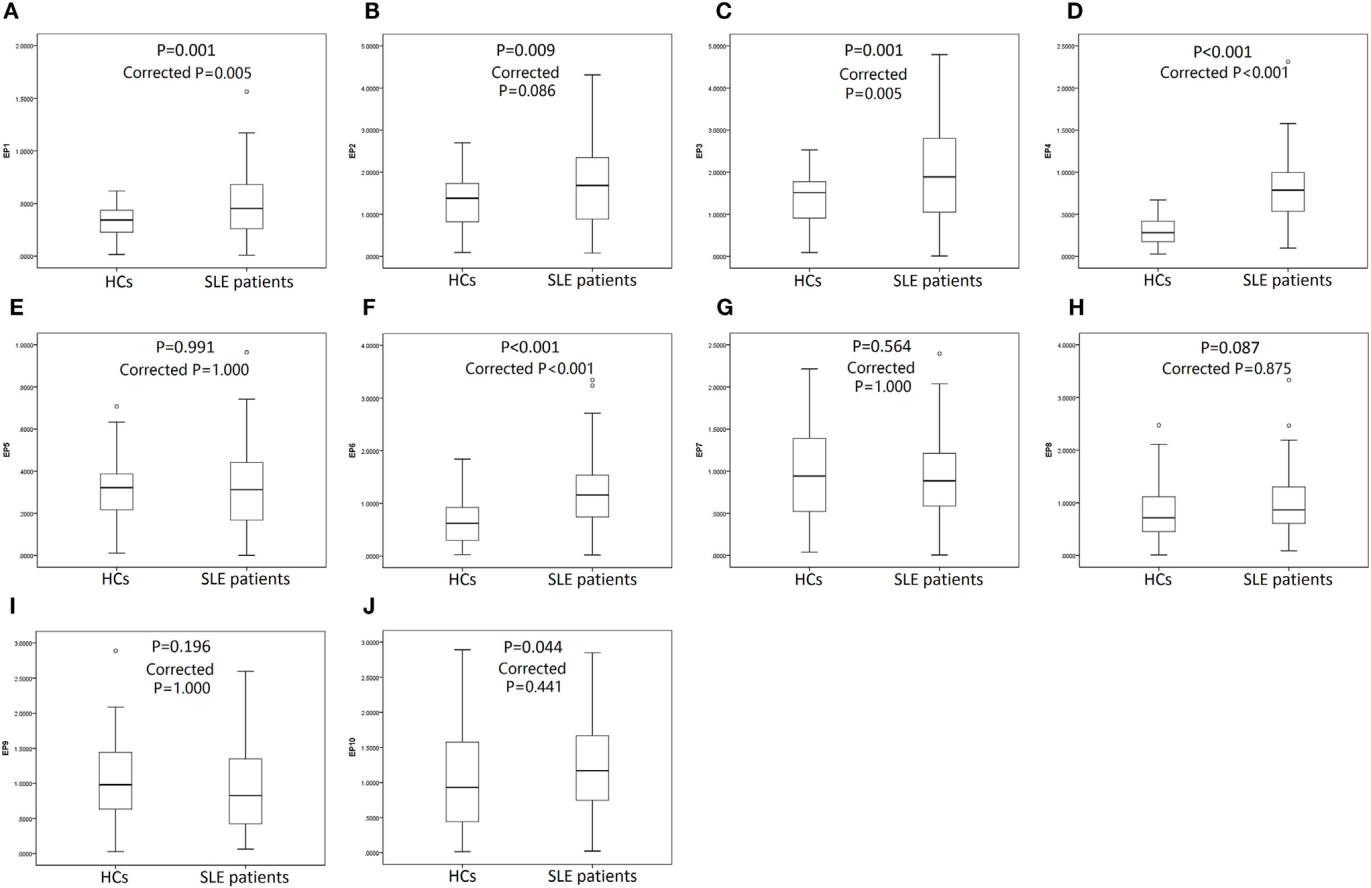
Serum EP1–10 levels in systemic lupus erythematosus (SLE) patients and health controls (HCs). SLE patients illuminated elevated levels of serum EP1 **(A)**, EP2 **(B)**, EP3 **(C)**, EP4 **(D)**, EP6 **(F)**, and EP10 **(J)** compared to HCs, while the levels were similar in EP5 **(E)**, EP7 **(G)**, EP8 **(H)**, and EP9 **(I)** between two groups. After Bonferroni correction, EP1 **(A)**, EP3 **(C)**, EP4 **(D)**, and EP6 **(F)** levels were higher in SLE patients compared with HCs, while the levels were similar in EP2 **(B)**, EP5 **(E)**, EP7–10 **(G–J)** between two groups. Comparison was determined by Wilcoxon rank sum test.

119 SLE patients with age 36.8 ± 11.5 years, 89% female were recruited. 64 HCs with matched age (35.2 ± 8.7 years, *P* = 0.332) and gender (84% female, *P* = 0.360) were also enrolled. The other detailed characteristics of SLE patients were presented in Table 3. As shown in Figure 4, SLE patients presented with a higher level of serum EP1 (*P* = 0.001, Figure 4A), EP2 (*P* = 0.009, Figure 4B), EP3 (*P* = 0.001, Figure 4C), EP4 (*P* < 0.001, Figure 4D), EP6 (*P* < 0.001, Figure 4F), and EP10 (*P* = 0.044, Figure 4J), while the levels were similar in EP5 (Figure 4E), EP7 (Figure 4G), EP8 (Figure 4H), and EP9 (Figure 4I) between two groups. In addition, after Bonferroni correction, EP1 (corrected *P* = 0.005, Figure 4A), EP3 (corrected *P* = 0.005, Figure 4C), EP4 (corrected *P* < 0.001, Figure 4D), and EP6 (corrected *P* < 0.001, Figure 4F) were disclosed to be increased in SLE patients compared with HCs, while no difference of EP2 (Figure 4B), EP5 (Figure 4E), and EP7–10 (Figures 4G–J) was discovered between two groups.

**Table 3:** Characteristics of SLE patients and HCs.

Items	SLE patients (*N* = 119)	HCs (*N* = 64)	*P*value
Age (years)	36.8 ± 11.5	35.2 ± 8.7	0.332
Gender (female *n*/%)	106 (89)	54 (84)	0.360
Disease duration (years)	8.5 (4.0–11.5)	–	–
Neurological disorder (*n*/%)	6 (5)	–	–
Renal involvement (*n*/%)	49 (41)	–	–
Arthritis (*n*/%)	21 (18)	–	–
Myocarditis (*n*/%)	5 (4)	–	–
Alopecia (*n*/%)	7 (6)	–	–
Erythra (*n*/%)	30 (25)	–	–
Ulcer (*n*/%)	7 (6)	–	–
Pleurisy (*n*/%)	2 (2)	–	–
Vasculitis (*n*/%)	1 (1)	–	–
Fever (*n*/%)	36 (30)	–	–
Thrombocytopenia (*n*/%)	15 (13)	–	–
Leukopenia (*n*/%)	28 (24)	–	–
Hematuria (*n*/%)	57 (48)	–	–
Proteinuria (*n*/%)	49 (41)	–	–
Cylindruria (*n*/%)	48 (40)	–	–
CRP (mg/L)	7.36 (3.47–18.60)	–	–
ESR (mm/h)	32.10 (9.46–44.77)	–	–
lgG (mg/mL)	14.26 (10.72–20.93)	–	–
lgA (mg/mL)	2.48 (1.52–3.95)	–	–
lgM (mg/mL)	0.99 (0.60–1.62)	–	–
ANA Positive (*n*/%)	110 (92)	–	–
Anti-dsDNA Positive (*n*/%)	59 (50)	–	–
Anti-SSA Positive (*n*/%)	64 (54)	–	–
Anti SSB Positive (*n*/%)	29 (24)	–	–
Anti-Sm Positive (*n*/%)	31 (26)	–	–
Anti u1RNP Positive (*n*/%)	55 (46)	–	–
Anti SCL70 Positive (*n*/%)	5 (4)	–	–
Anti Rib-*P* Positive (*n*/%)	63 (53)	–	–
SLEDAI Score	4.00 (2.00–7.00)	–	–

*Data were presented as mean ± SD, median (1/4–3/4), or count (%). Comparison was determined by Student test or Chi-square test. *P* < 0.05 was considered significant*.

*SLE, systemic lupus erythematosus; HCs, health controls; CRP, C-reactive protein; ESR, erythrocyte sedimentation rate; Ig, immunoglobulin; ANA, autoantibody to nuclear antigen; SSA, Sjogren’s syndrome A; SSB, Sjogren’s syndrome B; Sm B, Smith B; RNP, ribonucleoprotein; SCL70, scleroderma 70; Rib-P, anti-ribosomal p protein; SLEDAI, SLE disease activity index*.

**Figure 5:**
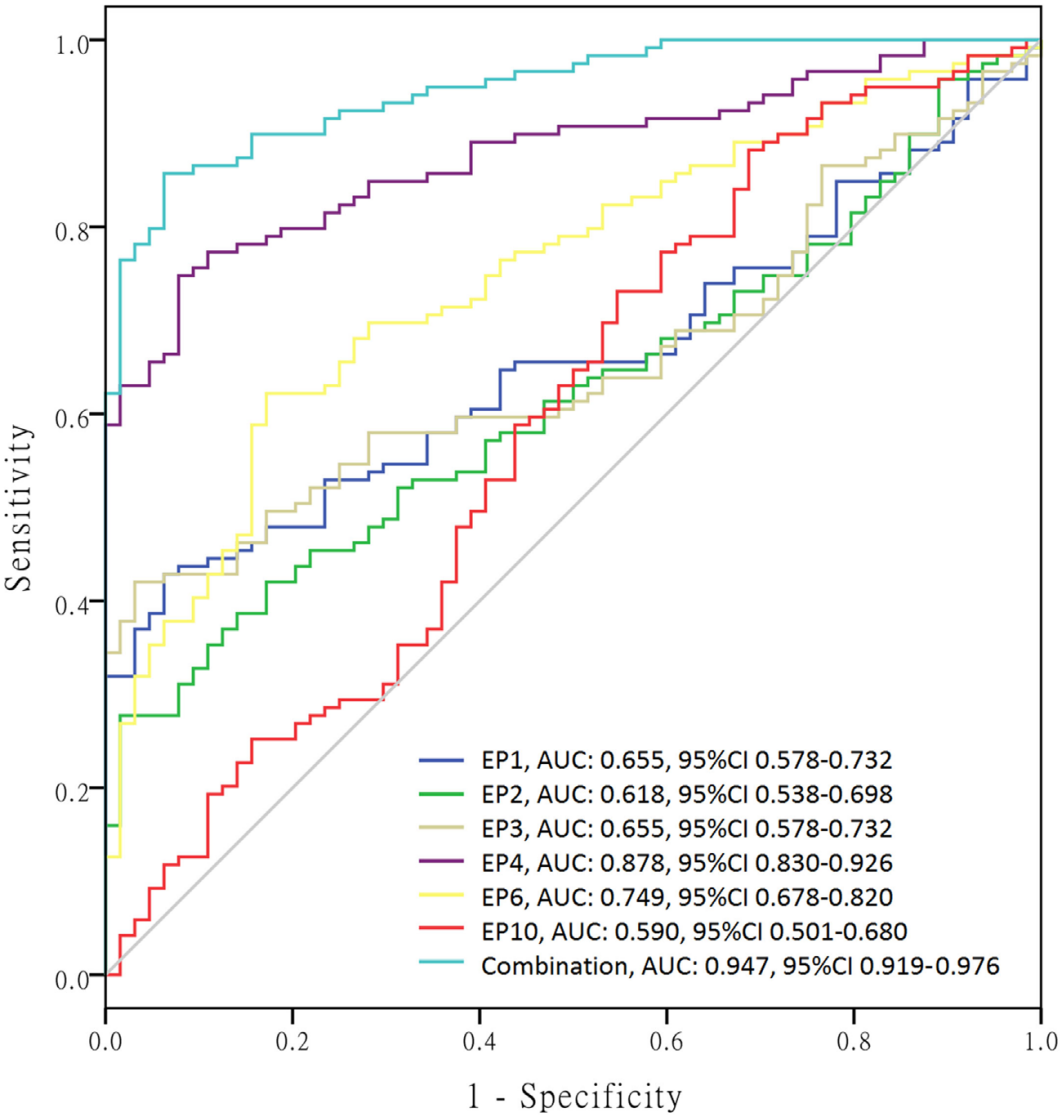
Receiver operating characteristic (ROC) curves of differently expressed epitope peptides (EPs) for systemic lupus erythematosus (SLE) diagnosis. ROC curves were drawn to explore the value of differently expressed EPs in predicting SLE risk. EP1–EP4, EP6, and EP10 revealed corresponding areas under curve (AUCs) as follows: EP1, AUC: 0.655, 95% CI 0.578–0.732; EP2, AUC: 0.618, 95% CI 0.538–0.698; EP3, AUC: 0.655, 95% CI 0.578–0.732; EP4, AUC: 0.878, 95% CI 0.830–0.926; EP6, AUC 0.749, 95% CI 0.678–0.820; EP10, AUC 0.590, 95% CI 0.501–0.680. More importantly, combination of EP1–EP4, EP6, and EP10 disclosed even better predicting value for SLE risk with AUC: 0.947, 95% CI 0.919–0.976.

In order to further investigate the value of differently expressed EPs in predicting SLE risk, ROC curves were performed (Figure 5) and we found that EP1–4, EP6, and EP10 levels were all good predictors for SLE susceptibility with area under curve (AUC) as follows: EP1, AUC: 0.655, 95% CI 0.578–0.732; EP2, AUC: 0.618, 95% CI 0.538–0.698; EP3, AUC: 0.655, 95% CI 0.578–0.732; EP4, AUC: 0.878, 95% CI 0.830–0.926; EP6, AUC 0.749, 95% CI 0.678–0.820; EP10, AUC 0.590, 95% CI 0.501–0.680. And when combined, these six parameters (EP1–4, EP6, and EP10) together, the diagnostic value for SLE was even greater with AUC: 0.947, 95% CI 0.919–0.976.

Subsequently, we calculated the sensitivity, specificity, false negative rate, and false positive rate of each index for SLE risk at best cutoff point in each ROC curve (Table 4), which disclosed that EP4 presented a good diagnostic value with sensitivity 71.4%, specificity 93.7%, false negative rate 28.6%, and false positive rate 6.3%, so as EP6 with sensitivity 62.2%, specificity 82.8%, false negative rate 37.8% and false positive rate 17.2%. More importantly, Combination of EP1–4, EP6, and EP10 showed an even better diagnostic value with sensitivity 82.4%, specificity 98.4%, false negative rate 17.6%, and false positive rate 1.6%. The best cutoff point was defined as the point at which the value of sensitivity plus specificity reached the maximum in the ROC curve.

**Table 4:** Sensitivity, specificity, false negative rate, and false positive rate of differently expressed epitope peptides for systemic lupus erythematosus risk.

Parameters	EP1 (%)	EP2 (%)	EP3 (%)	EP4 (%)	EP6 (%)	EP10 (%)	Combination (%)
Sensitivity	38.7	27.7	42.0	71.4	62.2	88.2	82.4
Specificity	95.3	98.4	96.9	93.7	82.8	31.2	98.4
False negative rate	61.3	72.3	58.0	28.6	37.8	11.8	17.6
False positive rate	4.7	1.6	3.1	6.3	17.2	68.8	1.6

*Data were presented as percentage. The data were calculated based on the best cutoff point at which the value of sensitivity plus specificity reached the maximum in the receiver operating characteristic curve*.

### Correlation of EP1–10 Levels with Disease Severity According to SLEDAI Score

**Figure 6:**
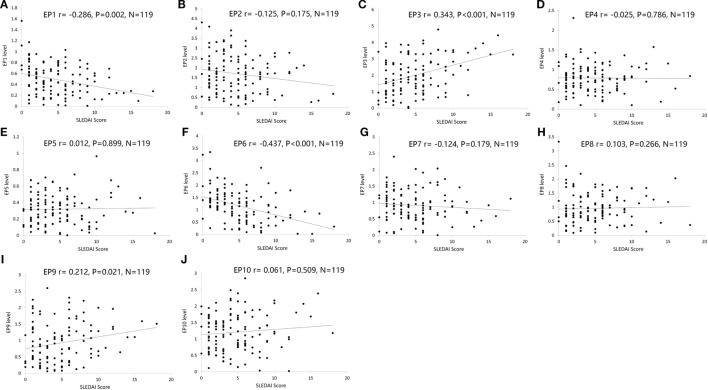
Correlation of EP1–10 levels with SLE disease activity index (SLEDAI) score. Spearman rank correlation test was performed to evaluate the association of serum EP1–10 levels with SLEDAI score, which illuminated that EP1 **(A)** and EP6 **(F)** levels were negatively correlated with SLEDAI score, while EP3 **(C)** and EP9 **(I)** levels were positively associated with SLEDAI score. No correlation of EP2, EP4, EP5, EP7, EP8, or EP10 levels with SLEDAI score was discovered **(B,D,E,G,H,J)**.

Furthermore, we explored the value of serum EP1–10 levels in disease severity management. As presented in Figure 6, EP1 (Figure 6A) and EP6 (Figure 6F) levels were negatively correlated with SLEDAI score (*P* = 0.002 and *P* < 0.001, respectively), while EP3 (Figure 6C) and EP9 (Figure 6I) levels were positively associated with SLEDAI score (*P* < 0.001 and *P* = 0.021, respectively). No correlations of EP2, EP4, EP5, EP7, EP8, and EP10 levels with SLEDAI score were observed (Figures 6B,D,E,G,H,J).

## Discussion

In our study, we disclosed that: (1) 10 probable EBV EA, MA, LMP-1, and LMP-2A B-cell epitopes related to SLE were predicted by analyzing amino acid sequences, secondary structures, transmembrane domains, surface properties, and homological comparison between the epitopes and SLE self-antigens. (2) EPs were combined and used to immunize the mice, and IgG concentration at week 8 were increased in all EPs groups (EP1–10) and KLH group compared with PBS group; while ANA levels by ELISA and ANA-positive rates by indirect immunofluorescence assay were both increased in only EP1, EP2, EP4, EP6, and EP10 groups compared to KLH group. (3) Indirect ELISA was performed to determine the levels of EP1–10 in serum of SLE patients and HCs, and we found EP1, EP2, EP3, EP4, EP6, and EP10 levels were increased in SLE patients compared with HCs, while after Bonferroni correction, only EP1, EP3, EP4, and EP6 levels were elevated. Furthermore, ROC curve showed a great diagnostic value of combination of EP1–4, EP6, and EP10 levels for predicting SLE risk. (4) EP1 and EP6 levels were negatively correlated with SLEDAI score, while EP3 and EP9 levels were positively associated with SLEDAI score.

Accumulating evidences disclose that EBV infection is associated with autoimmune diseases, including SLE, rheumatoid arthritis (RA), and multiple sclerosis (MS), which might result from both viral and immunological factors (6). Despite of the ambiguous mechanism of EBV infection in SLE pathogenesis, it is widely considered that the following factors might contribute to the consequence: (1) infection and immortalization of autoreactive B-cells, T-cells, and NK cells; (2) exacerbated inflammation by innate immune responses; (3) activation of HERVs related to autoimmunity; (4) cross-reactivity between microbial peptides and similar self-peptides caused by molecular mimicry; (5) augmenting autoimmunity through bystander activation such as promoting activation of autoreactive lymphocytes; (6) progressed autoimmunity through autoreactive T-cells escaping the negative selection allowed by dual T-cell receptor (19–25).

Molecular mimicry, characterized by cross-reactivity of B-cells, T cells and antibodies on account of sequential and/or structural similarities between virus and antigens that being first proposed at 1983, is one of the most critical causes of virus inducing autoimmunity, which leads to various autoimmune diseases including SLE, RA, MS, and so on (18, 26). Autoantibodies against epitopes on SmB and SmD are disclosed to illuminate cross-reactivity with various domains of EBNA-1, and EBNA-1 motif PPPGRRP immunized mice and rabbits present with lupus-like autoimmune disease (27–29). Besides, antibodies against Ro (169–180) (earliest detectable antiantibodies in SLE) cross-react with EBNA-1 as well, and rabbits immunized by the corresponding peptide disclose SLE-like symptoms including leukopenia, renal dysfunction, and thrombocytopenia (39). These indicate that based on the mechanism of molecular mimicry, EBV infection acts as crucial role in SLE pathogenesis through cross-reactivity between EBV and SLE self-antigens (40). However, few study investigating cross-reactivity of EBV EA, MA, LMP-1, and LMP-2A with B-cells in SLE is reported, which might explain a novel mechanism on how EBV infection causing SLE.

B-cell epitopes, classified into linear or conformational types, are important for understanding the antigenic structures and interactions of virus antigen–antibody at molecular dimension (41). Although multiple procedures have been introduced for mapping B-cell epitopes, including homolog-scanning mutagenesis, proteolysis of antigen–antibody complexes, region-specified PCR mutagenesis, and so on, the great improvement of technology in recent decades allows us to identify B-cell epitopes with dramatically decreased number of targeted proteins and according laboratory experiments based on various software and databases (42–44). Thus, in this present study, we predicted 48 possible EBV EA, MA, LMP-1, and LMP-2A B-cell epitopes through analyzing secondary structures, transmembrane domains, and surface properties by various methods based on the software and international databases. Subsequently the homological comparison was performed between EBV proteins and SLE self-antigens, including Sm B, Sm D, Sm E, rRNP, and Ro, by BLAST module. And 10 SLE self-antigens related EBV B-cell epitopes were identified named as epitope 1 to epitope 10 in our study. Interestingly, we found the candidate EBV epitopes had intermediate similarity rates instead of high similarity rates with SLE self-antigens peptides (mostly between 40 and 70%), thus we further analyzed the features of other different amino acids by investigating the hydrophilicity and acid-base property, and increased percentages of amino acids with similar characteristics were observed (mostly from 60 to 90%), which indicated the good homology between candidate EBV protein epitopes and SLE self-antigens. Then according peptides (EP1–10) were combined and purified to further investigate the role of EBV EA, MA, LMP-1, and LMP-2A B-cell epitopes in SLE pathogenesis.

Early antigen protein D, as an EBV lytic cycle antigen, is localized both in the cytoplasm and in the nucleus of infected cells. EBV EA binds dsDNA without sequence specificity and is essential for polymerase to replicate viral genome (32). MA, a leading vaccine candidate of EBV which is synthesized in the lytic cycle of infection, plays a key role during merozoite invasion into erythrocytes by interacting with Rhoptry Neck Protein 2 (RON2) (45, 46). LMP-1 and LMP-2A, as members of LMPs, function in EBV latent state of the infection. *in vitro* studies, LMP-1 is disclosed to be a protein that normally results from CD40 signal transduction pathway activated by CD4^+^ T-cells, and LMP-2A mimics a constitutively activated B-cell receptor. And LMP-1 as well as LMP-2A induce infected B-cells into GC process and assist EBV to entry the memory B-cell pool (6, 31, 47). In addition, LMP-1 is reported to activate the expression of IFN-α in EBV-infected B-cells and upregulates B-cell activating factors (BAFF), which induces the immunity (48). While LMP-2A prevents induction of energy in autoreactive B-cells and bring about bypass of tolerance checkpoints, which results in high expression of autoantibodies and inducing development of lupus-like disease (49). These suggest the essential role of EA, MA, LMP-1, and LMP-2A in EBV and in inducing immunity.

In our study, we immunized the mice with EP1–10 bounded with KLH and we found IgG levels were increased in EP1–10 immunization group compare to PBS controls, which indicated the immunogenicity of the EPs. As to ANA level, we observed that it was elevated in only EP1, EP2, EP3, EP4, EP6, EP7, and EP10 immunized groups compared with KLH control group by ELISA, while its positive rate was increased in only EP1, EP2, EP4, EP6, and EP10 groups by indirect immunofluorescence assay, which indicated the antigenicity of the EPs. Further measurement of anti-ENAs antibodies, including anti-SmB, anti-SmD, anti-SmE, anti-rRNP and anti SAA/Ro antibodies, in mice serum by ELISA also supported that EP1, EP4, EP6, and EP10 increased the levels of autoantibodies related to SLE. These suggest EBV might involve in the SLE development and progression through cross-reactivity of EBV EA (FEVSPDAVAEWQNHQ-Epitope 1, YKRPQGCS-Epitope 2, PPPPPRTP-Epitope 4), LMP-1 (LVSGAGDGPP-Epitope 6), and LMP-2A (GLALSL-Epitope 10) B-cell epitopes with SLE self-antigens.

In addition, serum samples from SLE patients and HCs were obtained, subsequently indirect ELISA was performed and we found serum EP1, EP2, EP3, EP4, EP6, and EP10 levels were increased in SLE patients compared with HCs, while after Bonferroni correction, only EP1, EP3, EP4, and EP6 levels were elevated. Furthermore, combination of serum EP1–4, EP6, and EP10 levels presented a great diagnostic value of SLE from HCs. These results were in line with the mice experiment which disclosed that EP1, EP4, EP6, and EP10 increased levels of ANA and anti-ENAs antibodies. At last, we also analyzed the correlation of serum EPs levels with disease severity (SLEDAI score) in SLE patients, and EP1, EP3, EP6, and EP9 were illuminated to be correlated with SLEDAI score. As to the consistency about the correlations of EPs with SLE risk and disease severity, EP1 and EP6 presented with good consistencies between predicting SLE risk and disease severity; however, EP4 and EP 10 presented a diagnostic value but not disease severity marker, while EP3 and EP9 correlated with disease severity but not SLE risk, the possible explanation were that: SLE was diagnosed by reaching 4 or above items out of 11 items according to ACR criteria for the classification of SLE which included not only the critical manifestations but also ANA dysregulation and abnormality of immunology, while SLEDAI score was mainly affected by critical manifestations; in this study, most of patients were diagnosed as SLE with ANA positive (92%), anti-dsDNA positive (50%), and anti-Sm positive (26%), and these accounted a lot in SLE diagnosis, thus the manifestations contributed relatively less to SLE diagnosis but contributed greatly to SLEDAI score; and we found EP4 and EP10 correlated with ANA positive and dsDNA positive greatly but not significantly associated with the critical manifestations with high weight (high score) in SLEDAI scales such as neurological disorder, vasculitis, and arthritis, while EP3 and EP9 presented the opposite results. These above-mentioned data implied EBV EA (FEVSPDAVAEWQNHQ-Epitope 1, PPPPRTP-Epitope 4), LMP-1 (LVSGAGDGPP-Epitope 6), LMP-2A (GLALSL-Epitope 10) B-cell epitopes might contribute to diagnosis of SLE and while EBV EA (FEVSPDAVAEWQNHQ-Epitope 1, PPPPPRTP-Epitope 4), LMP-1 (LVSGAGDGPP-Epitope 6), and LMP-2A (PGGLGTLGAA-Epitope 9) B-cell epitopes were conductive to the management of disease severity in some extents.

In conclusion, EBV EA, MA, LMP-1, and LMP-2A B-cell EPs increased SLE related autoantibodies in mice, and their indirect levels might be served as potential biomarkers for SLE diagnosis and disease severity.

## Author Contributions

JT and XW performed the research, designed the research study, analyzed the data, and wrote the paper. XW conceived and supervised the study. JT, XW, and GG performed the research. XX, XL, and XZ analyzed the data. LS designed the research and supervised the study. All the authors read and approved the final manuscript.

## Conflict of Interest Statement

The authors declare that the research was conducted in the absence of any commercial or financial relationships that could be construed as a potential conflict of interest.

## Footnotes

^1^http://www.uniprot.org/.

^2^http://www.dnastar.com.

^3^http://www.expasy.ch/tools.

^4^http://web.expasy.org/protscale/.

## Abbreviations

EBV, Epstein–Barr virus; EA, early antigen protein D; MA, envelope glycoprotein GP340/membrane antigen; LMP, latent membrane protein; SLE, systemic lupus erythematosus; ANA, autoantibody to nuclear antigen; HCs, health controls; SLEDAI, SLE disease activity index; EPs, epitope peptides; ELISA, enzyme-linked immunosorbent assay; ACR, American College of Rheumatology; AUC, area under curve.
