# Supplementary material for: The Possible Effect of B-Cell Epitopes of Epstein–Barr Virus Early Antigen, Membrane Antigen, Latent Membrane Protein-1, and -2A on Systemic Lupus Erythematosus
Source: Front Immunol. 2018 Feb 12;9:187. doi: 10.3389/fimmu.2018.00187 (PMC5819577; doi:10.3389/fimmu.2018.00187)
Supplement: Supplementary file 1 [file image_1.PDF]

Supplementary Material

The role of B-cell epitopes of Epstein-Barr virus early antigen, membrane antigen, latent membrane protein-1 and -2A in systemic lupus erythematosus

Jianxin Tu[1], Xiaobing Wang[1], Guannan Geng[2], Xiangyang Xue[3], Xiangyang Lin[4], Xiaochun Zhu[1] and Li Sun[1*]

***Correspondence:** Li Sun, Department of Rheumatology, The First Affiliated Hospital of Wenzhou Medical University, 2 Fuxue Street, Wenzhou 325000, China.

email: grassandsun@163.com

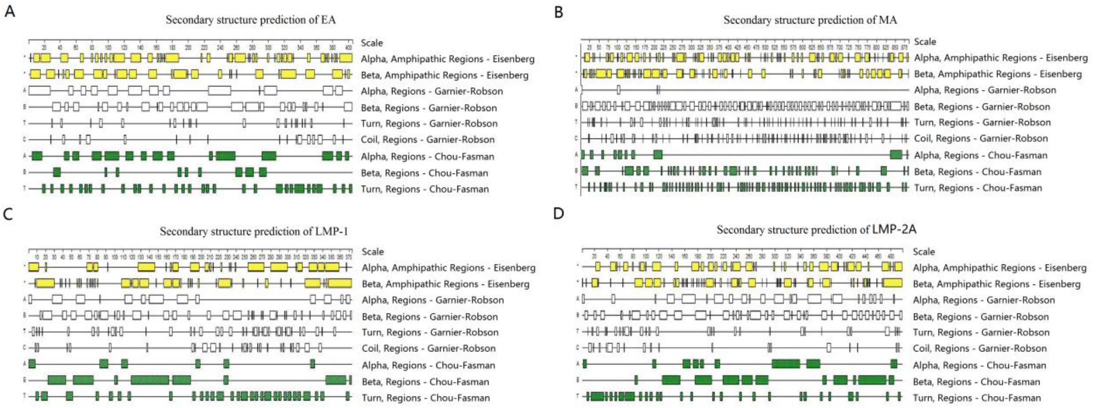

Supplemental Figure S1. Predication of second structure of EBV EA, MA, LMP-1 and LMP-2A by different methods. Amphiphilicity-Eisenberg, Secondary structure-Garnier-Robson and Secondary structure-Chou-Fasman methods of Protean module in DNAStar software (http://www.dnastar.com) were performed to analyze the second structure of EBV EA, MA, LMP-1 and LMP-2A. EBV EA mainly consisted of β sheets, followed by α helixes, and some inconstant coils as well as β turns (A), EBV MA mostly contained β sheets, followed by inconstant coils, β turns, and fewer α helixes (B), EBV LMP-1 mainly included β sheets, followed by α helixes, β turns and inconstant coils (C), EBV LMP-2A was mainly made up by α helixes, followed by inconstant coils, and some β sheets as well as β turns (D).
