# Supplementary material for: The Possible Effect of B-Cell Epitopes of Epstein–Barr Virus Early Antigen, Membrane Antigen, Latent Membrane Protein-1, and -2A on Systemic Lupus Erythematosus
Source: Front Immunol. 2018 Feb 12;9:187. doi: 10.3389/fimmu.2018.00187 (PMC5819577; doi:10.3389/fimmu.2018.00187)
Supplement: Supplementary file 2 [file image_2.PDF]

Supplementary Material

The role of B-cell epitopes of Epstein-Barr virus early antigen, membrane antigen, latent membrane protein-1 and -2A in systemic lupus erythematosus

Jianxin Tu[1], Xiaobing Wang[1], Guannan Geng[2], Xiangyang Xue[3], Xiangyang Lin[4], Xiaochun Zhu[1] and Li Sun[1*]

***Correspondence:** Li Sun, Department of Rheumatology, The First Affiliated Hospital of Wenzhou Medical University, 2 Fuxue Street, Wenzhou 325000, China.

email: grassandsun@163.com

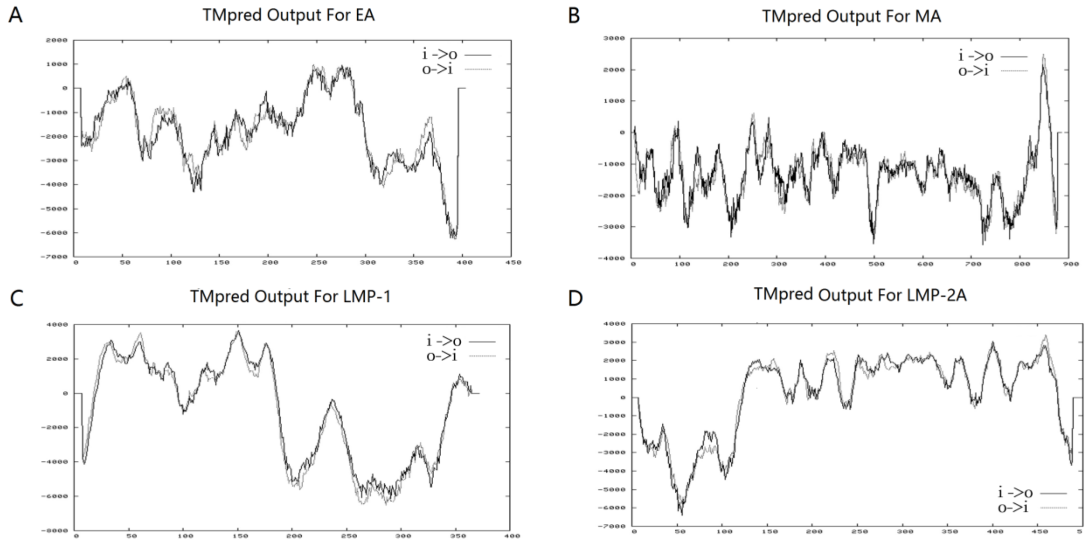

Supplemental Figure S2. Prediction of transmembrane domains of EBV EA, MA, LMP-1 and LMP-2A. Transmembrane domains were analyzed by using TMpred module on EXPASY Internet Server (http://www.expasy.ch/tools). EBV-EA presented 3 predicted transmembrane domains (A), EBV MA presented 6 predicted transmembrane domains (B), EBV LMP-1 presented 7 predicted transmembrane domains (C) and EBV LMP-2A presented 10 predicted transmembrane domains (D).
