# Supplementary material for: The Possible Effect of B-Cell Epitopes of Epstein–Barr Virus Early Antigen, Membrane Antigen, Latent Membrane Protein-1, and -2A on Systemic Lupus Erythematosus
Source: Front Immunol. 2018 Feb 12;9:187. doi: 10.3389/fimmu.2018.00187 (PMC5819577; doi:10.3389/fimmu.2018.00187)
Supplement: Supplementary file 3 [file image_3.PDF]

Supplementary Material

The role of B-cell epitopes of Epstein-Barr virus early antigen, membrane antigen, latent membrane protein-1 and -2A in systemic lupus erythematosus

Jianxin Tu[1], Xiaobing Wang[1], Guannan Geng[2], Xiangyang Xue[3], Xiangyang Lin[4], Xiaochun Zhu[1] and Li Sun[1*]

***Correspondence:** Li Sun, Department of Rheumatology, The First Affiliated Hospital of Wenzhou Medical University, 2 Fuxue Street, Wenzhou 325000, China.
email: grassandsun@163.com

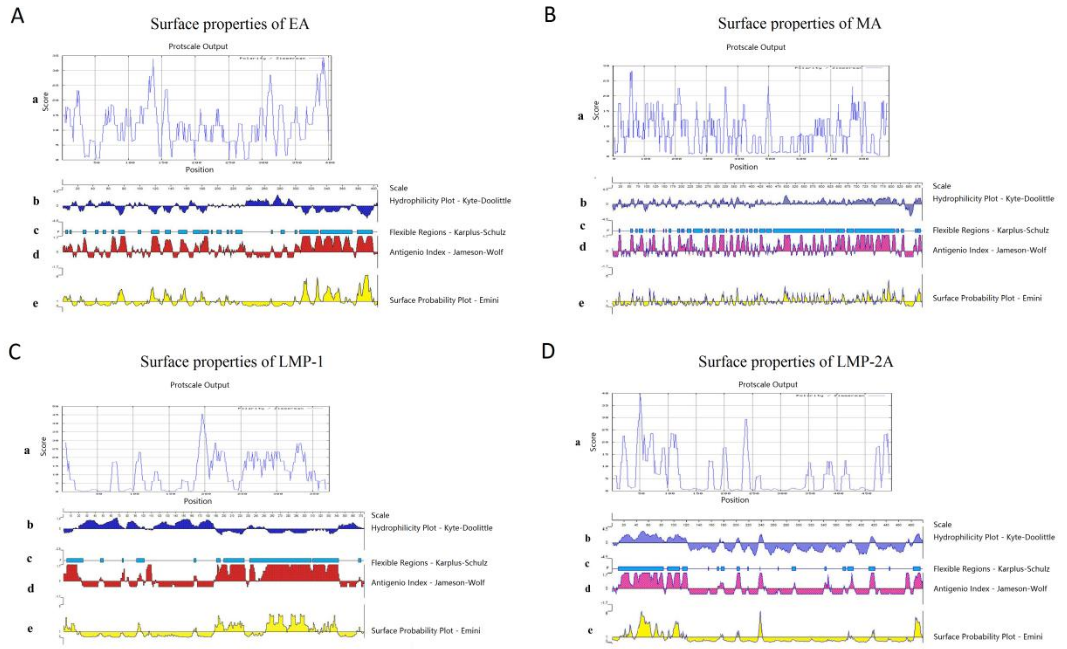

Supplemental Figure S3. Surface properties of EBV EA, MA, LMP-1 and LMP-2A. Surface probability-Emini, Flexibility-Karplus-Schulz, Hydropathy-Kyte-Doolittle, Antigenicity-Jameson-Wolf methods in DNAStar software (http://www.dnastar.com) and Palarity/Zimmerman method on EXPASY Internet Server (web.expasy.org/protscale/) were used to analyze the surface properties of EBV EA (A), MA (B), LMP-1 (C) and LMP-2A (D). The first line (a) of each image presented polarity parameter, the second line (b) of each image presented hydrophilicity parameter, the third line (c) of each image presented flexibility parameter, the fourth line (d) of each image presented antigenicity parameter, the fifth line (e) of each image presented probability parameter.
